# Psychological distress, financial literacy, loneliness, and coping in relation to financial difficulties: a cross-sectional study in Sweden

**DOI:** 10.1186/s12889-026-28309-w

**Published:** 2026-06-25

**Authors:** Elsa Willborg, Johanna Larsson, Jonas Berge, Sara Lindström, Anders Håkansson, Richard Ahlström, Henrik Levinsson

**Affiliations:** 1 Department of Psychology, Lund University, Lund, Sweden; 2 Faculty of Medicine, Department of Clinical Sciences Lund, Psychiatry, Lund University, Lund, Sweden; 3 Skåne University Hospital, Malmö Addiction Center, Malmö, Sweden; 4 Department of Psychiatry, Skåne University Hospital, Lund, Sweden

**Keywords:** Financial literacy, Psychological distress, Loneliness, Coping, Financial difficulties

## Abstract

**Background:**

Many individuals experience recurring or anticipated difficulties in meeting essential financial obligations, such as paying bills. Financial difficulties are associated with a range of adverse outcomes and are influenced not only by objective economic conditions but also by psychological factors, including psychological distress, loneliness, coping strategies, and financial literacy, which may shape how individuals perceive, manage, and respond to their financial situation. Against this background, it is important to further examine the economic and psychological factors associated with financial difficulties.

**Method:**

The present cross-sectional study examined whether financial literacy, psychological distress, loneliness, and coping were associated with financial difficulties, operationalised as recurring difficulties paying bills during the past 12 months and expected difficulties paying bills in the upcoming two months. Data was collected through an online questionnaire completed by 2,035 adults aged 18–96 during the autumn of 2024. Logistic regression analyses were conducted to identify variables independently associated with financial difficulties when considered simultaneously.

**Results:**

The analyses showed that financial literacy, psychological distress, loneliness, and avoidant coping were independently associated with both outcomes.

**Conclusions:**

This study examines contemporaneous associations between economic and psychological variables and self-reported financial difficulties. The cross-sectional design precludes conclusions regarding causality. Further longitudinal research is needed to clarify the direction and nature of these relationships.

**Supplementary Information:**

The online version contains supplementary material available at 10.1186/s12889-026-28309-w.

## Background

There is limited understanding of how key psychological, social, and behavioral factors jointly relate to financial difficulties. Although financial literacy, psychological distress, loneliness, and coping strategies have each been linked to financial difficulties, they have predominantly been studied in isolation. This has resulted in a fragmented evidence base in which the relative importance and interrelations of these factors remain unclear. Consequently, it is not well established which of these factors are independently associated with financial difficulties, and to what extent observed associations reflect shared variance across psychological, social, and behavioral domains. The factors were selected because they represent complementary domains that may jointly shape how individuals experience and manage financial strain, including financial knowledge (financial literacy), mental health (psychological distress), social relationships (loneliness), and coping-related behavioral strategies. This perspective reflects the need to distinguish key correlates of financial difficulties from overlapping influences across domains.

Addressing this gap requires analytical approaches that consider these factors simultaneously, in order to clarify their relative and independent associations. Improved understanding of how these factors co-occur may contribute to a more coherent evidence base for future research and potential applied work in this area.

A growing body of research has examined the associations between financial difficulties and health and well-being. Financial difficulties and over-indebtedness have been associated with poorer mental, physical, cognitive, and social health across adulthood [1–3] as well as with suicidal behavior [4–7]. Moreover, financial problems have been linked to increased loneliness [8, 9], and coping strategies, particularly avoidant coping, have been associated with poorer financial decision-making [10–12].

Based on existing literature on financial problems and the complex conditions surrounding the phenomenon, it appears important to deepen our understanding of how personal financial problems and indebtedness co-vary with both economic and psychological factors.

Financial difficulties can be understood in relation to individual differences in financial knowledge. In the early 2000s, a research field emerged focusing on individuals’ understanding of basic financial concepts. Anna-Maria Lusardi and colleagues, who have been central to this research, named this phenomenon financial literacy. The term refers to an individual’s ability to understand and apply financial knowledge in order to manage personal finances effectively. A substantial body of research has demonstrated strong associations between financial literacy and financial outcomes [13, 14]. Higher financial literacy has been linked to greater retirement saving [15, 16], lower levels of indebtedness, and a greater ability to manage financial shocks [17].

Despite its importance, financial literacy remains low globally. Across studies and populations, between one-third and one-half of adults fail to correctly answer questions about basic financial concepts such as interest rates, inflation, and risk diversification [14, 18, 19]. The link between financial literacy and various personal financial outcomes is well established and is also associated with demographic and socioeconomic factors such as gender, age, and income [13, 14, 20].

A robust association between psychological distress and financial difficulties has been documented in several review articles [2, 3, 6, 21]. Recently, a large U.S.-based review including 316 studies found strong associations between financial problems and psychological distress, as well as physical, cognitive, and functional health outcomes [2].

Ridley et al. [22] describe the relationship between psychological distress and financial difficulties as bidirectional and likely causal. Psychological distress may reduce labor market participation and income, while cognitive impairments associated with conditions such as depression may contribute to avoidant behavior and poorer financial decision-making. Conversely, negative financial shocks increase the risk of psychological distress, while poverty-reduction interventions, such as cash transfers, have been shown to improve mental health and reduce suicide risk. Interventions targeting psychological distress have also been found to produce positive economic outcomes. Together, these findings suggest that financial difficulties and psychological distress reinforce one another, forming self-perpetuating cycles that may also be interrupted through effective intervention.

Loneliness has likewise been shown to be associated with financial difficulties [1, 8]. Much of the existing research has focused on older adults [23–26]. For example, Cheung et al. [9] found that financial difficulties were among the strongest predictors of loneliness among older adults in New Zealand. However, associations have been observed across the adult life span. In a longitudinal study of adults in the United Kingdom, higher levels of financial difficulties predicted increases in loneliness over time [8]. Loneliness also mediated the relationship between financial difficulties and psychological distress, accounting for approximately 15% of the total effect. Gender moderated this association, with loneliness exerting a stronger influence on women’s psychological distress than on men’s. Overall, the relationship between loneliness and financial difficulties appears complex and influenced by multiple interacting factors.

According to the transactional model of stress and coping, introduced by Lazarus and Folkman [27], stress occurs when external demands are appraised as exceeding an individual’s available resources. Coping is defined as the cognitive and behavioral efforts used to manage these demands and is commonly divided into problem-focused coping, which targets the stressor itself, and emotion-focused coping, which regulates emotional responses. Subsequent research identified several commonly used strategies that fail to reduce negative affect or external stressors; these are now referred to as maladaptive coping and include behaviors such as self-criticism, avoidance, and substance use [28–32].

Research on coping and personal finances has primarily examined coping as a predictor or moderator of mental health among individuals struggling with their finances. Maladaptive coping strategies and emotion-focused coping have been associated with higher levels of psychological distress among indebted individuals in Sweden [11], as well as among economically disadvantaged populations in the United States [33] and Portugal following the financial crisis of 2011–2014 [34].

Fewer studies have examined direct associations between coping and financial outcomes. Some evidence suggests that problem-focused coping is associated with more adaptive long-term financial behaviors [12], although this research has largely focused on young adults. In a review, Jalili et al. [35] found that problem-solving strategies, such as income diversification and expenditure reduction, were protective for financial well-being. Caplan and Schooler [36] reported that lower socioeconomic status was associated with greater use of emotion-focused coping and less use of problem-focused coping, mediated by lower self-efficacy and higher fatalism. A perceived lack of control over one’s financial situation may therefore promote unhelpful coping strategies that further deteriorate personal finances.

Avoidant coping appears to be particularly detrimental for financial outcomes. Yi and Baumgartner [10] showed that feelings of shame following impulsive purchases predicted greater use of avoidant coping, which in turn increased the likelihood of repeated impulsive buying. Avoidant coping was also associated with poorer mental health, higher levels of credit debt, and lower rates of debt repayment. Supporting this, Hilbert et al. [37] found that financial difficulties predicted increased avoidance of information pertaining to personal finances over time, while avoidance of information in turn predicted greater financial scarcity.

### Aim and research questions

The aim of the present study was to examine whether psychological distress, coping strategies, loneliness and financial literacy were associated with personal financial difficulties among adults in Sweden. A secondary aim was to identify which among these variables showed independent associations to financial difficulties, when considered simultaneously. Gender, age, and income were included as control variables.

1. Are psychological distress, loneliness, coping, and financial literacy associated with
  - difficulties paying bills during the past 12 months?
  - expectations of difficulties paying bills during the coming two months?
2. When psychological distress, loneliness, coping strategies, and financial literacy are considered simultaneously, are they independently associated with
  - difficulties paying bills during the past 12 months, controlling for gender, age, and income?
  - expected difficulties paying bills during the next two months, controlling for gender, age, and income?

## Methods

### Design and procedure

Data were collected in Sweden during a period of economic downturn. According to a report by the Swedish Financial Supervisory Authority [38], prolonged periods of high interest rates and inflation have strained household finances, leaving many individuals with limited financial margins. Approximately one in three households reported difficulties in covering recurring expenses such as food and rent over the past 12 months [39]. This cross-sectional study was based on data which were collected over a four-week period in October and November 2024 using an online survey administered by Origo Group. The survey was sent to Origo Group’s web panel, which has over 100,000 panel members. Quotas for gender, age, and geographic region were applied to approximate the Swedish population distribution on these parameters. Before completing the survey, participants were provided with information about the study and its purpose, and were informed that participation was anonymous, voluntary, and could be withdrawn at any time. The survey was designed so that it could only be submitted once all questions were answered, resulting in no incomplete surveys in the study. Although forced completion eliminated item nonresponse, it may have affected data quality by encouraging satisficing behaviors, where participants provide less thoughtful responses to complete the survey. Mandatory responses may also have contributed to participant dropout before survey submission. The survey consisted of questions measuring financial literacy, personal financial situation, coping, loneliness, and mental health. Participants were also asked to provide information on a number of demographic variables, including age, gender, household composition, income, education and employment status.

### Participants

The survey was opened by 2,679 individuals. The final sample consisted of 2,035 participants indicating a completion rate of 76%. Participants ranged in age between 18 and 96 years, with a mean age of 52 years. In total, 1,022 participants were women and 998 were men, while 15 participants reported a gender other than female or male. See Table 1 for demographic data. For clarity, income categories have been compressed into three groups. This grouping roughly reflects the median salary in Sweden as well as the threshold for national income tax [40, 41].

**Table 1:** Demographic and socioeconomic characteristics of participants (*N* = 2035)

Characteristics	*n*	%	M (SD)	Min–max
Gender				
Woman	1022	50.2		
Man	998	49.0		
Other / Prefer not to say	15	0.7		
Age			51.96 (17.60)	18–96
Highest completed education				
Primary school or equivalent	145	7.1		
Upper secondary school	625	30.7		
Higher Vocational Education	323	15.9		
University/College	942	46.3		
Household composition				
Living with spouse/partner and children	544	26.7		
Living with spouse/partner without children	661	32.5		
Single parent with children living at home	140	6.9		
Single, without children living at home	614	30.2		
Living with parents	76	3.7		
Employment status				
Employed full-time/part-time	1078	53.0		
Student	103	5.1		
Unemployed	167	8.2		
Retired	552	27.1		
Disability/Activity benefits	148	7.3		
Other	68	3.3		
Monthly gross income				
Low (< 25,000 SEK)	850	42.0		
Medium (25 000–50 000 SEK)	971	48.0		
High (> 50 000 SEK)	214	11.0		

For employment status, participants could select multiple responses. approximate conversion: 1 Euro (EUR) ≈ 11.5 SEK

*SEK* Swedish kronor

## Methods and materials

In the present study, financial difficulties were used as the outcome measure. Previous research has measured financial difficulties in a variety of ways. The Swedish Enforcement Authority [42] describes three established methods to measure over-indebtedness. The administrative method relies on official records of payment difficulties, such as record of non-payment, bankruptcies, and applications for debt restructuring. However, this method underestimates over-indebtedness, as not all affected individuals are recorded in formal debt resolution procedures. The objective method calculates household debt burdens in relation to income and assets. It provides population-level averages across households but does not identify how many individual households experience financial distress, as debt and resources are unevenly distributed. The subjective method is the one applied by the Swedish Enforcement Authority [42] and measures the amount of over-indebted households from the household’s own subjective view of the possibilities to pay off their debt. The obvious weakness with this method is the subjectiveness, although it also opens up the possibility of capturing households who would normally be missed with other measures, for example those with loans to private individuals. This study opted for a similar definition and used two subjective questions to capture persistent financial difficulties over the past year, as well as concerns about financial difficulties in the near future. This approach is consistent with prior research and official reports [42, 43] and allows for the identification of financially vulnerable individuals who may not be captured in administrative records. While brief, such subjective indicators have been shown to reflect meaningful aspects of financial difficulties, particularly in population-based surveys where objective financial data are not available.

The questions focused on the participants’ personal financial situation and could be answered with the response options “Yes” or “No.” The first question was: “In the past 12 months, have you experienced that you (or you and others living in your household) have recurring problems paying your bills?” and the second question was: “Do you expect that you (or you and others living in your household) will have large problems paying your bills during the next two months?” The retrospective time span represents a sufficiently long period for the difficulties to be considered persistent, whereas the prospective time span represents a period for which it is reasonable to expect that a person with stable finances would have a plan.

### Financial literacy

To measure financial literacy, as described in our previous study [12], the respondents were asked to answer three questions regarding their financial knowledge with the ‘Big Three’ measure. The ‘Big Three’ measure was created by Lusardi and Mitchell [44] and measures the respondent’s financial literacy through four key principles: simplicity, relevance, brevity, and capacity to differentiate. When designing the measure, they identified three basic economic principles that people should have some understanding of; these are (i) understanding of interest compounding; (ii) understanding of inflation, and (iii) understanding of risk-diversification [44]. Included in the ‘Big Three’ measure are the questions:

1. Suppose you had SEK 100 in a savings account and the interest rate was 2% per year. After 5 years, how much do you think you would have in the account if you left the money to grow?
2. Imagine that the interest rate on your savings account was 1% per year and inflation was 2% per year. After 1 year, how much would you be able to buy with the money in this account?
3. Please tell me whether this statement is true or false. ‘Buying a single company’s stock usually provides a safer return than a stock mutual fund’.

All questions are multiple-choice, with only one correct answer. Each question includes the option “Do not know”, which is considered a lack of financial literacy. A high score on the ‘Big Three’ questions is associated with savvy financial behavior, in for instance, retirement planning and debt management, and could therefore be indicative of financial well-being. Lusardi argues that the “Big Three” concern such fundamental concepts that one needs to answer all questions correctly in order to be considered financially literate [44]. Accordingly, answering all three questions correctly was used as the threshold for financial literacy, whereas answering only one or two correctly was considered insufficient. This dichotomization facilitates comparison with previous studies. The scale has been implemented in several surveys, such as the National Longitudinal Survey of Youth (NLSY), the National Financial Capability Study (NFCS), and the Survey of Consumer Finances (SCF), to name a few [16]. This scale has also been used in other published studies by the authors [12, 45]. The Swedish version of the ‘Big Three’ questions was taken from a report by the Swedish Financial Supervisory Authority [46].

### Psychological distress

In this study, psychological distress was operationalized as self-reported symptoms of depression and anxiety and measured using two rating scales: the Generalized Anxiety Disorder-7 (GAD-7) for anxiety and the Patient Health Questionnaire-9 (PHQ-9) for depression. The GAD-7 assesses how often participants have experienced various problems over the past 14 days. Responses are given on a four-point Likert scale with the options “not at all,” “several days,” “more than half the days,” and “nearly every day.” Possible total scores on the scale range from 0 to 21. For screening for anxiety disorders, a cut-off score of 10 or higher is recommended [47].

The PHQ-9 assesses how often respondents have experienced depressive symptoms during the past 14 days. Responses are given on a four-point Likert scale with the options “not at all,” “several days,” “more than half the days,” and “nearly every day.” Possible total scores on the scale range from 0 to 27. According to Kroenke et al. [48], scores of 10 or higher could indicate major depressive disorder.

Although depression and anxiety are conceptually distinct clinical constructs, the PHQ-9 and GAD-7 were highly correlated in the present study (Pearson’s *r* = .87), raising concerns about multicollinearity in regression models including both measures simultaneously (VIF = 4.02, tolerance = 0.25). To address this, we conducted an exploratory factor analysis (EFA) to evaluate whether the items from both scales could be represented by a single underlying distress factor.

EFA was performed in R using the psych package, with maximum likelihood estimation and oblimin rotation. Suitability of the data for factor analysis was confirmed prior to extraction. Parallel analysis and inspection of the scree plot supported retention of a single factor. The one-factor solution explained 61% of the variance (SS loadings = 9.82), with all item loadings ranging from 0.69 to 0.86, indicating strong and uniform item-factor relationships. Internal consistency of the combined 16-item solution was excellent (Cronbach’s α = 0.96). Factor scores were computed using the regression method (Thurstone), yielding a continuous score for each participant, with higher scores reflecting greater overall psychological distress.

To further evaluate the appropriateness of combining the two scales, we also fitted one-factor solutions to each scale independently. The GAD-7 showed good unidimensional fit (RMSEA = 0.093, TLI = 0.969, RMSR = 0.03, α = 0.94), while the PHQ-9 showed poorer fit as a unidimensional scale (RMSEA = 0.123, TLI = 0.910, RMSR = 0.05, α = 0.93), consistent with prior literature suggesting a more complex factor structure for the PHQ-9. The combined 16-item solution (RMSEA = 0.108, TLI = 0.896, RMSR = 0.05) performed comparably to or better than the PHQ-9 alone across fit indices, supporting the decision to use a composite distress measure rather than retaining the scales separately. Although RMSEA values exceeded the conventional threshold of 0.06 across all solutions, this is common with large samples and ordinal item-level data, and the remaining fit indicators alongside the strong loadings and high internal consistency support the interpretability and reliability of the composite score.

### Loneliness

To measure loneliness, the UCLA Loneliness Scale, Version 3, was used [49]. The scale consists of 20 items answered on a four-point Likert scale with the response options “Never,” “Rarely,” “Sometimes,” and “Always.” The Swedish translation was conducted by Käll et al. [50]. In the present sample internal consistency for the scale was excellent with Cronbach’s α = 0.92.

### Coping

To measure the respondents’ coping behavior, as described in our previous study [12], respondents were asked to answer the BriefCOPE inventory. The BriefCOPE was developed in 1997 as a shortened version of the 60-item ‘Coping Orientation to Problems Experienced Inventory’ by Carver et al. [28] and instead features 28 items measuring the coping style of the respondent [51]. The Swedish translation of the instrument was made by Muhonen and Torkelson [52]. In 2012, Dias et al. [53] conducted a psychometric analysis of the measure and divided it into three subscales: problem-focused coping, emotion-focused coping, and avoidant coping. This categorisation was used in the present study. The three subscales showed acceptable to good internal consistency with Cronbach’s α = 0.83 for problem-focused coping, α = 0.75 for emotion-focused coping, and α = 0.83 for avoidant coping.

The results are interpreted based on the sum of all items within each category. For example, the sum of all questions regarding avoidant coping = level of avoidant coping. The maximum number of points to be scored for the problem-focused and avoidant categorizations is 32 points and 48 for the emotion-focused categorization.

### Age, gender, and income

Participants were asked the question, “What year were you born?” and were instructed to indicate their birth year. To collect data on gender, participants could select one of four response options: “Female,” “Male,” “Other, namely:”, or “Prefer not to say.” To obtain information on participants’ income, the question “What is your current monthly income before taxes?” with the following response categories: less than 10,000 SEK; 10,000–15,000 SEK; 15,000–20,000 SEK; 20,000–25,000 SEK; 25,000–30,000 SEK; 30,000–35,000 SEK; 35,000–40,000 SEK; 40,000–45,000 SEK; 45,000–50,000 SEK; 50,000–70,000 SEK; and 70,000 SEK or more (1 EUR ≈ 11.5 SEK). For legibility, a condensed categorization is presented in Table 1. All analyses were conducted using the full scale above, which was treated as ordinal.

### Data analysis

Statistical analyses were conducted using IBM SPSS Statistics 29 [54]. The significance level was set at α = 0.05. For the gender variable, a total of 15 participants had indicated either ‘Other’ or ‘Prefer not to say’. These groups were too small to yield statistically meaningful results and were therefore coded as missing values in all analyses, leaving only the categories “Female” and “Male”.

Prior to conducting the analyses, the assumptions were examined. Boxplots were inspected to identify potential outliers. Comparisons between observed means and 5% trimmed means indicated no substantial influence of extreme values; all cases were retained. Skewness and kurtosis values ranged between − 1 and + 1 for all variables, indicating no substantial deviations from normality. Based on these distributional properties, Pearson’s correlation coefficient was used to assess bivariate associations. Prior to model estimation, multicollinearity diagnostics were examined. All independent variables, except GAD-7 and PHQ-9 (see EFA section), showed acceptable variance inflation factor (VIF) and tolerance values, indicating no evidence of problematic multicollinearity.

To address Research Question 1, a correlation analysis was conducted using Pearsons’s correlation coefficient. For relationships between categorical variables, the Phi coefficient was used.

The second research question was analyzed using hierarchical logistic regression. The outcome variables, financial literacy and gender were recoded to create a reference group with a value of 0 and a comparison group with a value of 1. For the two financial outcome variables, the reference group consisted of participants who answered ‘No’ to the questions, and thus did not report financial problems. For financial literacy, the reference group consisted of participants considered to be lacking financial literacy. For gender, the reference group consisted of women. Linearity in the logit for continuous variables was assessed using the Box-Tidwell procedure. Age was modelled using both linear and quadratic terms due to consistent indications of non-linearity in diagnostic tests for the association with difficulties paying bills over the past 12 months. For all other continuous predictors, including coping variables, isolated indications of non-linearity were observed in preliminary Box-Tidwell tests. However, these did not result in any meaningful changes in model fit or effect estimates in the fully adjusted analyses and were therefore retained in their linear form to ensure parsimony and interpretability. For the outcome measuring difficulties paying bills during the next two months, age was modelled in its linear form, as no consistent indications of non-linearity were detected in the Box-Tidwell procedure.

For Research Question 2, gender, age, and income were first entered as control variables to assess whether adding the variables of interest improved the model fit and explained variance. The remaining variables were entered in separate blocks. Financial literacy was entered in Block 2, followed by psychological distress, coping, and loneliness. Odds ratios (ORs) were used to indicate the increase in the outcome variable associated with a one-unit change in the predictor. The interpretation of odds ratios (ORs) depends on the scale of the predictor. For continuous variables, a one-unit change may not be meaningful. Therefore, ORs were also calculated for one standard deviation change to provide a more interpretable measure of the change in odds. Odds ratios per standard deviation were derived using unrounded regression coefficients and standard deviations. Psychological distress was standardized prior to analysis. Accordingly, the reported odds ratio represents the change in the odds of the outcome associated with a one-standard-deviation increase in psychological distress.

### Ethical considerations

This study was conducted in accordance with the Declaration of Helsinki. An application was submitted to the Swedish Ethical Review Authority. The application was dismissed (Dnr 2024-04044-01) since the study involved no interventions on participants and no processing of personal data. The Authority provided the following ethical advisory statement: “The Ethical Review Authority has no ethical objections to the research project”.

Informed consent was obtained before accessing the survey. No personal data was collected by either the research team or Origo Group, ensuring anonymity. The survey included potentially sensitive questions on mental health and one’s financial situation, as stated in the study information. To address possible negative consequences, participants were referred to healthcare services at the end of the survey in case of distress, and those in need of debt management were referred to the municipal budget and debt counseling services.

### Use of AI tools

ChatGPT (OpenAI) was used to assist with language editing, including improvements to clarity, flow, and grammar of the manuscript. The authors reviewed and take responsibility for all final content.

## Results

Table 2 presents descriptive statistics for the studied variables. A relatively large proportion of the sample had experienced financial difficulties: approximately 24% reported that they had experienced recurring problems paying their bills during the past 12 months. One fifth of the respondents expected large difficulties in paying their bills in the coming two months. In our sample, approximately 46% answered all three questions on financial literacy correctly. This implies that more than half (approximately 54%) lack financial literacy according to Lusardi’s definition [44]. The question most frequently answered correctly concerned interest rates, with 87% identifying the correct answer, while 71% answered the inflation question correctly and only 59% answered the risk diversification question correctly. For distribution of correct answers see Table 3.

**Table 2:** Descriptive statistics for financial literacy, psychological distress, loneliness, coping and financial difficulties (*N* = 2035)

Variable	*n*	%	M (SD)	Min-max
Financial literacy				
Financially literate	927	45.6		
Lacks financial literacy	1108	54.4		
Total	2035	100		
GAD-7	2035	100	5.54 (5.74)	0–21
PHQ-9	2035	100	6.83 (6.80)	0–27
Loneliness	2035	100	44.42 (11.64)	20–78
Problem-focused coping	2035	100	21.55 (4.95)	8–32
Emotion-focused coping	2035	100	28.36 (5.96)	12–48
Avoidant coping	2035	100	14.77 (4.96)	8–32
Problems paying bills in the past 12 months				
Yes	484	23.8		
No	1551	76.2		
Total	2035	100		
Expecting problems paying bills in next two months				
Yes	416	20.4		
No	1619	79.6		
Total	2035	100		

**Table 3:** Distribution of financial literacy correct answers

Financial literacy correct answers	*n*		%
0		112	5.5
1		355	17.4
2		641	31.5
3		927	45.6
Total		2035	100.0

### Research question 1

Table 4 presents the zero-order correlations between the independent variables and the two outcome variables. Previous difficulties paying bills were negatively associated with financial literacy and positively associated with psychological distress, loneliness, emotion-focused coping, and avoidant coping. A small negative association was observed with problem-focused coping. Similarly, expected difficulties paying bills were negatively associated with financial literacy and positively associated with psychological distress, loneliness, emotion-focused coping, and avoidant coping, with a small negative association for problem-focused coping.

**Table 4:** Correlation coefficients between the study variables (*N* = 2020)

Variable	1	2	3	4	5	6	7	8
1. Financial literacy	—							
2. Psychological distress	− 0.26***	—						
3. Loneliness	− 0.20***	0.63***	—					
4. Problem-focused coping	0.10***	− 0.12***	− 0.35***	—				
5. Emotion-focused coping	− 0.03	0.22***	− 0.06**	0.62***	—			
6. Avoidant coping	− 0.24***	0.64***	0.53***	0.03	0.38***	—		
7. Previous difficulties paying bills	− 0.21***	0.48***	0.35***	− 0.06*	0.11***	0.36***	—	
8. Expected difficulties paying bills	− 0.20***	0.49***	0.35***	− 0.05*	0.13***	0.38***	N/A	—

*N/A* Not applicable

*p ≤ .05, **p ≤ .01, ***p≤ .001

### Research question 2

The final model for the outcome variable *having experienced difficulties paying bills* is presented in Table 5. The Block 1 model, which included only control variables, was statistically significant, χ²(4) = 314.36, *p* < .001, Nagelkerke *R*² = 0.22. Age and income showed independent association with the outcome variable, while gender did not.

**Table 5:** Logistic regression of self-reported difficulties paying bills during the past 12 months (*N* = 2020)

Variable	B	Wald	*p*	OR	95% CI lower	95% CI upper
Financial literacy	-0.47	11.64	< 0.001	0.63	0.48	0.82
Psychological distress	0.69	63.10	< 0.001	1.99	1.68	2.36
Problem-focused coping	0.02	0.97	0.326	1.02	0.98	1.05
Emotion-focused coping	-0.01	0.28	0.597	0.99	0.96	1.02
Avoidant coping	0.05	6.88	0.009	1.05	1.01	1.08
Loneliness	0.02	6.94	0.008	1.02	1.01	1.04
Gender	0.27	4.32	0.038	1.31	1.02	1.70
Monthly gross income	-0.15	35.61	< 0.001	0.86	0.82	0.90
Age	0.11	17.21	< 0.001	1.11	1.06	1.17
Age²	-0.00	23.43	< 0.001	1.00	1.00	1.00
Intercept	-4.18	26.72	< 0.001	0.02	—	—

Reference categories: gender = women; financial literacy = lacking financial literacy

*OR* odds ratio, *CI* confidence interval

The final regression model was statistically significant, χ²(10) = 565,93, *p* < .001, Nagelkerke *R*² = 0.37. The Hosmer-Lemeshow goodness-of-fit-test was significant (*p* = .029) however, this is common in large samples. The corresponding contingency table was inspected to assess whether the discrepancy reflected a systematic pattern of miscalibration. No systematic deviations between observed and expected frequencies were identified. Discriminative ability was assessed using the area under the receiver operating characteristic curve (AUC), based on the predicted probabilities from the logistic regression model. The model showed good discrimination, AUC = 0.84, *p* < .001, 95% CI [0.82, 0.86].

Psychological distress was independently associated with past difficulties paying bills, see Table 5. For each one-unit increase in the psychological distress factor score, the odds of having experienced difficulties paying bills increased by 99%. Financial literacy also showed an independent association. In the final model, individuals lacking financial literacy had 59% higher odds of having experienced difficulties paying bills compared to those with financial literacy. Loneliness and avoidant coping were also independently associated with the outcome variable. For each one-point increase on the loneliness scale, the odds of having experienced difficulties paying bills increased by 2%; an increase of one standard deviation corresponded to a 26% increase in odds. For each one-point increase in avoidant coping, the odds increased by 5%; an increase of one standard deviation corresponded to a 27% increase in odds. Problem-focused coping and emotion-focused coping showed no significant associations in the final model. Robustness checks using GAD-7 and PHQ-9 separately yielded highly similar results (see Supplementary Table A5 & A6). Robustness checks using GAD-7 and PHQ-9 separately produced results that were highly consistent with the main model. For the GAD-7 model χ²(10) = 553.72, *p* < .001, Nagelkerke *R²* = 0.36, and depressive symptoms in the PHQ-9 model, χ²(10) = 559.87, *p* < .001, Nagelkerke *R²* = 0.36, were both positively associated with previous difficulties paying bills. In both models, financial literacy, avoidant coping, loneliness, age, and monthly gross income remained statistically significant, whereas problem-focused coping, emotion-focused coping, and gender did not. Similarly, a robustness check treating financial literacy as a continuous score from 0 to 3 yielded results consistent with the main model. Financial literacy remained significantly associated with lower odds of having had difficulties paying bills during the past 12 months, B = -0.21, OR = 0.81, 95% CI [0.71, 0.92], *p* = .002. The overall model was statistically significant, χ²(10) = 563.77, *p* < .001, Nagelkerke *R²* = 0.37. Full estimates are provided in Supplementary Table A7. Further robustness checks were conducted using education, household composition, and employment status as additional controls. All previously significant variables of interest remained independently associated with the outcome. See Supplementary Table A8.

The final model for the outcome variable *expecting difficulties paying bills during the next two months* is presented in Table 6. The final regression model was statistically significant, χ²(9) = 544,51, *p* < .001, Nagelkerke *R*² = 0.37. The Hosmer-Lemeshow goodness-of-fit-test was significant (*p* = .007). No systematic deviations between observed and expected frequencies were identified in the corresponding contingency table. Discriminative ability was assessed using the area under the receiver operating characteristic curve (AUC), based on the predicted probabilities from the logistic regression model. The model showed good discrimination, AUC = 0.85, *p* < .001, 95% CI [0.83, 0.87].

**Table 6:** Logistic regression of self-reported expected difficulties paying bills during the next two months (*N* = 2020)

Variable	B	Wald	*p*	OR	95% CI lower	95% CI upper
Financial literacy	-0.42	8.13	0.004	0.66	0.49	0.88
Psychological distress	0.79	76.87	< 0.001	2.20	1.85	2.63
Problem-focused coping	0.02	1.60	0.207	1.02	0.99	1.06
Emotion-focused coping	-0.01	0.73	0.392	0.99	0.96	1.02
Avoidant coping	0.06	10.59	0.001	1.06	1.02	1.10
Loneliness	0.02	6.84	0.009	1.02	1.01	1.04
Gender	0.19	1.90	0.168	1.21	0.92	1.59
Monthly gross income	-0.12	20.83	< 0.001	0.88	0.84	0.93
Age	-0.02	13.52	< 0.001	0.98	0.98	0.99
Intercept	-2.26	11.06	< 0.001	0.11	—	—

Reference categories: gender = women; financial literacy = lacking financial literacy

*OR* odds ratio, *CI* confidence interval

Psychological distress also showed an independent association with expected difficulties paying bills. For each one-unit increase in the psychological distress factor score, the odds of expecting difficulties paying bills increased by 120%. Financial literacy showed an independent association with the outcome variable as well. In the final model, individuals who lacked financial literacy had 52% higher odds of expecting difficulties paying bills. Loneliness and avoidant coping also remained independently associated with the outcome variable. For each one-point increase on the loneliness scale, the odds of expecting difficulties paying bills increased by 2%; an increase of one standard deviation corresponded to a 29% increase in odds. For each one-point increase in avoidant coping, the odds increased by 6%; an increase of one standard deviation corresponded to a 34% increase in odds. For expected difficulties paying bills during the next two months, robustness checks using GAD-7 and PHQ-9 separately produced results that were highly consistent with the main model. For the GAD-7 model χ²(9) = 532.15, *p* < .001, Nagelkerke *R²* = 0.36, and depressive symptoms in the PHQ-9 model, χ²(9) = 532.19, *p* < .001, Nagelkerke *R²* = 0.36, were both positively associated with previous difficulties paying bills. In both models, financial literacy, avoidant coping, loneliness, age, and monthly gross income remained statistically significant, whereas problem-focused coping, emotion-focused coping, and gender did not. Full estimates are reported in Supplementary Tables B5 and B6. Likewise, a robustness check treating financial literacy as a continuous score from 0 to 3 yielded results consistent with the main model. Higher financial literacy remained significantly associated with lower odds of the outcome, B = -0.15, OR = 0.86, 95% CI [0.75, 0.99], *p* = .034. The overall model was statistically significant, χ²(9) = 540.73, *p* < .001, Nagelkerke *R²* = 0.37. Full estimates are provided in Supplementary Table B7. A further robustness check was conducted using education, household composition, and employment status as additional controls. All previously significant variables of interest remained independently associated with the outcome. See Supplementary Table B8.

## Discussion

The study found correlations of varying magnitude between the variables of interest and the self-reported measures of financial difficulties. Problem-focused and emotion-focused coping showed only very weak correlations with financial difficulties and were not independently associated with the outcomes in the regression models. The remaining variables showed weak to moderate correlations with the outcome variables. Psychological distress demonstrated the strongest correlations, followed by avoidant coping and loneliness.

Financial literacy was independently associated with financial difficulties in both models even when controlling for gender, age, and income. Previous research lacks consensus on this issue. West et al. [55] reported that financial literacy was not significantly associated with financial well-being while income showed a strong association. The results of the present study do not fully support this finding. Although income is independently associated with financial difficulties, financial literacy consistently showed additional independent association with financial difficulties. This is more in line with findings reported by Balasubramanian and Sargent and Hu et al. [20, 56], both of whom found that financial literacy has a significant association with financial well-being even when controlling for variables such as income and age. While financial literacy appears to show a stable relationship to self-reported financial difficulties, the inclusion of psychological distress, loneliness, and coping significantly improved both models. The results suggest that financial literacy appears to be one of several factors associated with self-reported financial difficulties, but in itself an insufficient explanation for financial difficulties.

Psychological distress was found to have the strongest numerical association to both outcome variables. Individuals with higher levels of psychological distress had higher odds of experiencing persistent self-reported financial difficulties in our study. This link is also supported in the existing literature [2, 22]. Based on the present study, it is not possible to draw conclusions about the direction of the relationship. Previous research has found support for bidirectional relationships, or negative spirals. Ridley et al. [22] describe how poorer financial circumstances lead to poorer mental health, which in turn leads to lost income, increased healthcare costs, and poorer financial decision-making. Avoidant coping was also associated with higher odds of reporting financial difficulties in the model for expected difficulties paying bills. Again, it is not possible to infer causality from our findings. However, previous research also supports the existence of bidirectional relationships. For example, Hilbert et al. [37] reported in a longitudinal study that financial difficulties both predict and are predicted by avoidant coping.

We also found support that loneliness is associated with both past and anticipated, self-reported difficulties in paying bills. Research in this area remains limited and has predominantly focused on older populations [23–26]. However, earlier studies have demonstrated an association between poorer finances and higher levels of loneliness [8]. The direction of this relationship cannot be established, either in the present study or in previous research. Chai et al. [8], however, examined the relationship longitudinally and found that financial difficulties predict loneliness over time.

Perhaps more surprisingly, there were no clear associations between problem-focused coping and self-reported financial difficulties. Problem-focused coping was not significant in either of the regression models and showed only very weak correlations with the outcome measures. Given the size of our sample, these correlations likely do not reflect any meaningful effect in practice. Previous research has identified negative associations between problem-focused coping and lower socioeconomic status [36], as well as unfavorable financial behaviors such as taking out consumer loans among young adults [12]. One proposed theory has been that individuals who engage more frequently in problem-focused coping are more likely to make proactive and preventive financial decisions, which in turn become economically beneficial in the long term [12, 57]. The present study finds no clear evidence of an association between more constructive stress-management strategies and financial difficulties in the present sample. However, it is important to note that the outcome variables differ between the present study and, for example, Levinsson et al. [12]. Taking or not taking consumer loans is a relatively simple behavioral measure, whereas recurring difficulties in paying one’s bills can result from many factors, both within and beyond the individual. Thus, the results of the present study may also highlight the importance of understanding persistent financial difficulties as a complex and multifaceted problem that cannot be reduced to simple individual shortcomings or behaviors.

### Strengths and limitations

The study has several strengths. One strength is the large sample size, which was recruited through an online web panel and quota-matched to reflect the population in terms of gender, age, and geography. The measurement instruments used in the study are well established and demonstrate high reliability and validity. Income is a particularly important control variable, as there is good reason to assume that it correlates with an individual’s ability to pay bills. The fact that our variables of interest contributed information to the models even after controlling for income suggests that these variables are independently associated with financial difficulties beyond income. However, there are also certain limitations which should be considered as the results are interpreted. The primary limitation of this study is its cross-sectional design, which prevents conclusions about the direction of associations.

Recruitment via a web panel may have introduced some self-selection bias as individuals who choose to participate may systematically differ from those who do not (for example in terms of interest in or perceived personal relevance of the topic, psychological distress, or financial situation). As with all self-reported data, responses may also be consciously or unconsciously influenced by social desirability, particularly for sensitive questions about personal finances and mental health, although the anonymity of the survey may help mitigate this bias. In addition, the online format excludes individuals without adequate internet access or digital literacy, potentially excluding segments of the population with lower socioeconomic status or limited technological resources.

Financial literacy in our sample was lower than typically reported in Sweden: only 46% met the criteria for basic financial literacy (Table 2), compared with 65–70% in previous studies [18, 19, 58]. One plausible interpretation is that the sampling process may have resulted in an overrepresentation of individuals with self-reported financial difficulties, which may also be associated with lower levels of financial literacy. This pattern is consistent with the selection mechanisms outlined above and suggests that the sample may not be fully representative, thereby limiting external validity and warranting caution in generalizing the findings. These considerations should be taken into account when interpreting the observed associations in the present study.

Due to insufficient statistical power, individuals identifying outside the gender binary were excluded from the analyses. This was a methodological necessity. Future studies with larger or specifically designed samples are needed to adequately examine financial difficulties in gender-diverse populations.

The present study examined associations between general coping and self-reported financial difficulties. It is unclear whether general coping styles necessarily correspond to how individuals manage stress specifically related to financial matters. An alternative approach would have been to measure “financial coping,” defined as the strategies individuals or households use to manage economic demands perceived as exceeding their resources [36].

Further limitations concern the measurement of financial difficulties. The outcome variables were based on two self-reported binary items capturing recurring and anticipated problems paying bills. While this approach is consistent with definitions used in previous research and by the Swedish Enforcement Authority [42, 43], which emphasize subjective assessments to identify financially vulnerable individuals not captured in administrative data, it does not fully capture the multidimensional nature or severity of financial hardship. In contrast to multi-item or objective measures, the present indicators rely on individual perceptions and may be influenced by differences in interpretation or response tendencies. Future research would benefit from combining subjective and objective indicators, as well as multi-item measures, to provide a more comprehensive assessment of financial hardship.

The measures of financial difficulties were based on specific time frames: the past 12 months and the two upcoming months. Some temporal delimitation was necessary for the responses to be interpretable, and the two different time frames served different purposes. The retrospective time span represented a sufficiently long period for financial difficulties to be considered persistent, whereas the prospective time span reflected a period for which it is reasonable to expect that individuals with stable financial circumstances would have some degree of financial planning. However, it may be discussed whether alternative time frames would have provided a clearer picture of individuals’ financial situations. In addition, the variables and the financial difficulties measures did not always refer to the same temporal or situational context. Psychological distress was assessed over the past 14 days, whereas the financial difficulty measures referred to both retrospective and anticipated financial problems.

These differences may have attenuated or otherwise influenced the observed associations and should therefore be considered when interpreting the findings.

There are also possible confounding factors that were not controlled for in this study, such as marital status and psychiatric diagnoses.

### Practical implications and future research

The associations identified in the present study may have implications for how financial difficulties and psychological distress are understood in relation to each other. The most consistent association was observed between psychological distress and self-reported financial difficulties, suggesting that these variables are closely related in the present sample. This may be relevant for research and applied contexts where financial and psychological well-being are considered jointly.

The results of the present study also open up the possibility that interventions aimed at increasing individuals’ financial literacy may be associated with differences in personal financial difficulties. However, given the cross-sectional design, the present study cannot establish any causal relationships between financial literacy and self-reported financial difficulties. This would be consistent with previous research [59]. For example, financial education in schools could be one such intervention. A meta-analysis has shown that interventions aimed at increasing financial knowledge are generally effective in promoting beneficial financial behavior [60].

Based on the study’s findings, future research could focus on a deeper understanding of the relationships between psychological and personal financial variables. Several significant associations were found among the independent variables themselves, ranging from weak to strong. There is limited prior research on the relationship between financial literacy and psychological variables, and further investigation is needed to better understand these associations. Investigating whether financial literacy may play a role in the relationship between psychological distress and financial difficulties may represent a promising avenue for future research. It may also be of interest to investigate how these relationships differ across age groups. Longitudinal studies could provide further insight into temporal ordering and the relationships between personal finances and psychological variables.

## Conclusions

In this cross-sectional study, we found that financial literacy, psychological distress, loneliness, and avoidant coping are independently associated with self-reported financial difficulties among Swedish adults. However, a deeper understanding of the nature of these relationships is lacking, and further research is required to map the underlying mechanisms. The present study represents another building block in the highly topical field examining the interplay between psychology and personal finance, highlighting the importance of further research into how these domains are interrelated.

## Supplementary Information

Supplementary Material 1.

## Data Availability

The data presented in this study are available from the corresponding author upon reasonable request due to ethical restrictions. Although the data are anonymous, respondents were not informed in the information letter that their responses could be made publicly available. Therefore, for ethical reasons, the data are not publicly accessible.

## Abbreviations

EFA: Exploratory factor analysis
EUR: Euros
GAD: Generalized Anxiety Disorder
OECD: Organisation for Economic Co-operation and Development
N/A: Not applicable
NLSY: National Longitudinal Survey of Youth
NFCS: National Financial Capability Study
SCF: Survey of Consumer Finances
SEK: Swedish krona
PHQ: Patient Health Questionnaire
OR: Odds Ratio
