# Supplementary material for: Psychological distress, financial literacy, loneliness, and coping in relation to financial difficulties: a cross-sectional study in Sweden
Source: BMC Public Health. 2026 Jun 25;26:2124. doi: 10.1186/s12889-026-28309-w (PMC13359890; doi:10.1186/s12889-026-28309-w)
Supplement: Supplementary file 1 — Supplementary Material 1. [file 12889_2026_28309_MOESM1_ESM.docx]

**Supplemental material**

**Table A1**

*Logistic regression of self-reported difficulties paying bills during the past 12 months, Block 1 (N = 2020)*

| Variable | *B* | Wald | *p* | OR | 95% CI lower | 95% CI upper |
| --- | --- | --- | --- | --- | --- | --- |
| Gender | 0.190 | 2.65 | .103 | 1.21 | 0.96 | 1.52 |
| Monthly gross income | -0.241 | 113.42 | < .001 | 0.79 | 0.75 | 0.82 |
| Age | 0.116 | 24.03 | < .001 | 1.12 | 1.07 | 1.18 |
| Age² | -0.002 | 43.11 | <.001 | 1.00 | 1.00 | 1.00 |
| Intercept | -1.461 | 7.65 | .006 | 0.23 | — | — |

***Note.*** *OR = odds ratio; CI = confidence interval. Reference categories: gender = women. Age² denotes the quadratic age term included in the model.*

The Block 1 model, which included only control variables, was statistically significant, χ²(4) = 314.36, *p* < .001, Nagelkerke *R*² = .22, AIC = 1906.

**Table A2**

*Logistic regression of self-reported difficulties paying bills during the past 12 months, Block 2 (N = 2020)*

| Variable | *B* | Wald | *p* | OR | 95% CI lower | 95% CI upper |
| --- | --- | --- | --- | --- | --- | --- |
| Financial literacy | -0.685 | 29.46 | < .001 | 0.50 | 0.39 | 0.65 |
| Gender | 0.289 | 5.93 | .015 | 1.34 | 1.06 | 1.69 |
| Monthly gross income | -0.223 | 93.55 | < .001 | 0.80 | 0.77 | 0.84 |
| Age | 0.115 | 23.57 | < .001 | 1.12 | 1.07 | 1.18 |
| Age² | -0.002 | 41.01 | < .001 | 1.00 | 1.00 | 1.00 |
| Intercept | -1.400 | 6.95 | .008 | 0.25 | — | — |

***Note.*** *OR = odds ratio; CI = confidence interval. Reference categories: gender = women; financial literacy = lacking financial literacy. Age² denotes the quadratic age term included in the model*

The complete model for block 2 was statistically significant, χ²(5) = 344.78, *p* < .001, Nagelkerke *R*² = .24, AIC = 1878,

**Table A3**

*Logistic regression of self-reported difficulties paying bills during the past 12 months, Block 3 (N = 2020)*

| Variable | *B* | Wald | *p* | OR | 95% CI lower | 95% CI upper |
| --- | --- | --- | --- | --- | --- | --- |
| Financial literacy | -0.504 | 13.94 | < .001 | 0.60 | 0.46 | 0.79 |
| Psychological distress | 0.909 | 176.89 | < .001 | 2.48 | 2.17 | 2.84 |
| Gender | 0.336 | 6.91 | .009 | 1.40 | 1.09 | 1.80 |
| Monthly gross income | -0.157 | 38.80 | < .001 | 0.86 | 0.81 | 0.90 |
| Age | 0.102 | 16.85 | < .001 | 1.11 | 1.06 | 1.16 |
| Age² | -0.001 | 24.06 | < .001 | 1.00 | 1.00 | 1.00 |
| Intercept | -2.241 | 15.52 | < .001 | 0.11 | — | — |

***Note.*** *OR = odds ratio; CI = confidence interval. Reference categories: gender = women; financial literacy = lacking financial literacy. Age² denotes the quadratic age term included in the model*

The complete model for block 3 was statistically significant, χ²(6) = 547.00, *p* < .001, Nagelkerke *R*² = .36, AIC = 1678.

**Table A4**

*Logistic regression of self-reported difficulties paying bills during the past 12 months, Block 4 (N = 2020)*

| Variable | *B* | Wald | *p* | OR | 95% CI lower | 95% CI upper |
| --- | --- | --- | --- | --- | --- | --- |
| Financial literacy | -0.457 | 11.20 | < .001 | 0.63 | 0.49 | 0.83 |
| Psychological distress | 0.781 | 95.12 | < .001 | 2.18 | 1.87 | 2.55 |
| Problem-focused coping | 0.006 | 0.11 | .745 | 1.01 | 0.97 | 1.04 |
| Emotion-focused coping | -0.012 | 0.66 | .418 | 0.99 | 0.96 | 1.02 |
| Avoidant coping | 0.058 | 11.79 | < .001 | 1.06 | 1.03 | 1.10 |
| Gender | 0.269 | 4.22 | .040 | 1.31 | 1.01 | 1.69 |
| Monthly gross income | -0.161 | 48.38 | < .001 | 0.85 | 0.81 | 0.89 |
| Age | 0.112 | 19.69 | < .001 | 1.12 | 1.07 | 1.18 |
| Age² | -0.001 | 26.22 | < .001 | 1.00 | 1.00 | 1.00 |
| Intercept | -3.161 | 19.99 | < .001 | 0.04 | — | — |

***Note.*** *OR = odds ratio; CI = confidence interval. Reference categories: gender = women; financial literacy = lacking financial literacy. Age² denotes the quadratic age term included in the model*

The complete model for block 4 was statistically significant, χ²(9) = 559.02, *p* < .001, Nagelkerke *R*² = .36, AIC = 1672,

The final regression model was statistically significant, χ²(9) = 540,48, *p* < .001, Nagelkerke *R²* = .35, AIC = 1690.

**Table A5**

*Logistic regression of self-reported difficulties paying bills during the past 12 months with GAD-7 (N = 2020)*

| Variable | *B* | Wald | *p* | OR | Lower 95% CI | Upper 95% CI |
| --- | --- | --- | --- | --- | --- | --- |
| Anxiety symptoms (GAD-7) | 0.101 | 52.64 | < .001 | 1.11 | 1.08 | 1.14 |
| Financial literacy | -0.484 | 12.62 | < .001 | 0.62 | 0.47 | 0.81 |
| Problem-focused coping | 0.015 | 0.76 | .385 | 1.02 | 0.98 | 1.05 |
| Emotion-focused coping | -0.007 | 0.20 | .652 | 0.99 | 0.97 | 1.02 |
| Avoidant coping | 0.053 | 9.47 | .002 | 1.05 | 1.02 | 1.09 |
| Loneliness | 0.027 | 12.25 | < .001 | 1.03 | 1.01 | 1.04 |
| Gender | 0.234 | 3.25 | .071 | 1.26 | 0.98 | 1.63 |
| Monthly gross income | -0.157 | 37.74 | < .001 | 0.86 | 0.81 | 0.90 |
| Age | 0.107 | 18.03 | < .001 | 1.11 | 1.06 | 1.17 |
| Age² | -0.001 | 24.35 | < .001 | 1.00 | 1.00 | 1.00 |
| Intercept | -5.808 | 55.58 | < .001 | 0.003 | — | — |

***Note.*** *OR = odds ratio; CI = confidence interval. Reference categories: gender = women; financial literacy = lacking financial literacy. Age² denotes the quadratic age term included in the model.*

The regression model of self-reported difficulties paying bills during the past 12 months, containing only GAD-7 as a measurement of psychological distress, was statistically significant, χ²(10) = 553,72, *p* <.001, Nagelkerke *R²* = .36, AIC = 1679.

**Table A6**

*Logistic regression of self-reported difficulties paying bills during the past 12 months with PHQ-9 (N = 2020)*

| Variable | *B* | Wald | *p* | OR | Lower 95% CI | Upper 95% CI |
| --- | --- | --- | --- | --- | --- | --- |
| Depressive symptoms (PHQ-9) | 0.092 | 57.41 | < .001 | 1.10 | 1.07 | 1.12 |
| Financial literacy | -0.456 | 11.09 | < .001 | 0.63 | 0.49 | 0.83 |
| Problem-focused coping | 0.017 | 0.95 | .331 | 1.02 | 0.98 | 1.05 |
| Emotion-focused coping | -0.005 | 0.11 | .736 | 1.00 | 0.97 | 1.03 |
| Avoidant coping | 0.048 | 7.72 | .005 | 1.05 | 1.01 | 1.09 |
| Loneliness | 0.022 | 7.96 | .005 | 1.02 | 1.01 | 1.04 |
| Gender | 0.281 | 4.61 | .032 | 1.33 | 1.03 | 1.71 |
| Monthly gross income | -0.159 | 38.90 | < .001 | 0.85 | 0.81 | 0.90 |
| Age | 0.107 | 17.71 | < .001 | 1.11 | 1.06 | 1.17 |
| Age² | -0.001 | 24.63 | < .001 | 1.00 | 1.00 | 1.00 |
| Intercept | -5.759 | 54.54 | < .001 | 0.003 | — | — |

***Note.*** *OR = odds ratio; CI = confidence interval. Reference categories: gender = women; financial literacy = lacking financial literacy. Age² denotes the quadratic age term included in the model.*

The regression model of self-reported difficulties paying bills during the past 12 months, containing only PHQ-9 as a measurement of psychological distress, was statistically significant, χ²(10) = 559.87, *p* <.001, Nagelkerke *R²* = .36, AIC = 1673.

**Table A7**

*Logistic regression of self-reported difficulties paying bills during the past 12 months with financial literacy as a continuous variable (N = 2020)*

| Variable | *B* | Wald | *p* | OR | Lower 95% CI | Upper 95% CI |
| --- | --- | --- | --- | --- | --- | --- |
| Financial literacy continous | -0.213 | 9.66 | .002 | 0.81 | 0.71 | 0.92 |
| Psychological distress | 0.694 | 64.33 | < .001 | 2.00 | 1.69 | 2.37 |
| Problem-focused coping | 0.019 | 1.08 | .299 | 1.02 | 0.98 | 1.06 |
| Emotion-focused coping | -0.009 | 0.38 | .540 | 0.99 | 0.96 | 1.02 |
| Avoidant coping | 0.045 | 6.62 | .010 | 1.05 | 1.01 | 1.08 |
| Loneliness | 0.021 | 6.88 | .009 | 1.02 | 1.01 | 1.04 |
| Gender | 0.257 | 3.87 | .049 | 1.29 | 1.00 | 1.67 |
| Monthly gross income | -0.154 | 36.34 | < .001 | 0.86 | 0.82 | 0.90 |
| Age | 0.104 | 16.87 | < .001 | 1.11 | 1.06 | 1.17 |
| Age² | -0.001 | 22.91 | < .001 | 1.00 | 1.00 | 1.00 |
| Intercept | -3.86 | 22.40 | < .001 | 0.02 | — | — |

***Note.*** *OR = odds ratio; CI = confidence interval. Reference categories: gender = women; financial literacy = lacking financial literacy. Age² denotes the quadratic age term included in the model.*

The regression model of self-reported difficulties paying bills during the past 12 months, containing financial literacy as a continuous variable, was statistically significant, χ²(10) = 563.77, *p* <.001, Nagelkerke *R²* = .37, AIC = 1669.

**Table A8**

*Logistic regression of self-reported difficulties paying bills during the past 12 months with additional control variables (N = 2020)*

| Variable | *B* | Wald | *p* | OR | 95% CI Lower | 95% CI Upper |
| --- | --- | --- | --- | --- | --- | --- |
| Financial literacy | -0.420 | 8.69 | .003 | 0.66 | 0.50 | 0.87 |
| Psychological distress | 0.682 | 58.27 | < .001 | 1.98 | 1.66 | 2.36 |
| Problem-focused coping | 0.019 | 1.09 | .298 | 1.02 | 0.98 | 1.06 |
| Emotion-focused coping | -0.012 | 0.63 | .429 | 0.99 | 0.96 | 1.02 |
| Avoidant coping | 0.043 | 5.68 | .017 | 1.04 | 1.01 | 1.08 |
| Loneliness | 0.022 | 7.36 | .007 | 1.02 | 1.01 | 1.04 |
| Gender | 0.267 | 3.88 | .049 | 1.31 | 1.00 | 1.70 |
| Monthly gross income | -0.156 | 20.95 | < .001 | 0.86 | 0.80 | 0.92 |
| Age | 0.071 | 4.61 | .032 | 1.07 | 1.01 | 1.15 |
| Age² | -0.001 | 5.95 | .015 | 1.00 | 1.00 | 1.00 |
| Employed full-time/part-time | 0.416 | 1.70 | .193 | 1.52 | 0.81 | 2.84 |
| Student | 0.559 | 2.68 | .102 | 1.75 | 0.90 | 3.42 |
| Unemployed | 0.650 | 3.91 | .048 | 1.92 | 1.01 | 3.65 |
| Retired | 0.371 | 0.74 | .389 | 1.45 | 0.62 | 3.37 |
| Disability/activity benefits | 0.315 | 0.77 | .380 | 1.37 | 0.68 | 2.77 |
| Other employment status | 0.428 | 1.02 | .314 | 1.53 | 0.67 | 3.53 |
| Partner, no children | -0.438 | 5.05 | .025 | 0.65 | 0.44 | 0.95 |
| Single, no children | -0.289 | 2.82 | .093 | 0.75 | 0.53 | 1.05 |
| Single, with children | 0.617 | 6.73 | .010 | 1.85 | 1.16 | 2.95 |
| Living with parents | -0.933 | 7.28 | .007 | 0.39 | 0.20 | 0.78 |
| Upper secondary school | -0.307 | 1.42 | .233 | 0.74 | 0.44 | 1.22 |
| Higher vocational education | 0.198 | 0.50 | .478 | 1.22 | 0.71 | 2.11 |
| University/College | -0.522 | 3.86 | .049 | 0.59 | 0.35 | 1.00 |
| Intercept | -3.392 | 11.59 | < .001 | 0.03 | — | — |

***Note****. OR = odds ratio; CI = confidence interval. Reference categories: gender = women; financial literacy = lacking financial literacy; education = primary education; household composition = living with partner and children; employment status = option not chosen. Age² denotes the quadratic age term included in the model.*

The regression model for self-reported difficulties paying bills during the past 12 months, using household composition, education and employment status as additional controls was statistically significant, χ²(23) = 612.78, *p* < .001, Nagelkerke *R²* = .39, AIC = 1646.

**Table B1**

*Logistic regression of self-reported expected difficulties paying bills during the next two months, Block 1 (N = 2020)*

| Variable | *B* | Wald | *p* | OR | 95% CI lower | 95% CI upper |
| --- | --- | --- | --- | --- | --- | --- |
| Gender | 0.097 | .646 | .421 | 1.10 | 0.87 | 1.40 |
| Monthly gross income | -0.215 | 83.64 | < .001 | 0.81 | 0.77 | 0.85 |
| Age | -0.041 | 132.17 | < .001 | 0.96 | 0.95 | 0.97 |
| Intercept | 1.662 | 63.17 | < .001 | 5.27 | — | — |

***Note.*** *OR = odds ratio; CI = confidence interval. Reference group for gender = women.*

The Block 1 model, which included only control variables, was statistically significant, χ²(3) = 245.84, *p* < .001, Nagelkerke *R*² = .18, AIC = 1803.

**Table B2**

*Logistic regression of self-reported expected difficulties paying bills during the next two months, Block 2 (N = 2020)*

| Variable | *B* | Wald | *p* | OR | 95% CI lower | 95% CI upper |
| --- | --- | --- | --- | --- | --- | --- |
| Financial literacy | -0.703 | 27.92 | < .001 | 0.50 | 0.38 | 0.64 |
| Gender | 0.198 | 2.586 | .108 | 1.22 | 0.96 | 1.55 |
| Monthly gross income | -0.198 | 68.33 | < .001 | 0.82 | 0.78 | 0.86 |
| Age | -0.039 | 111.97 | < .001 | 0.96 | 0.96 | 0.97 |
| Intercept | 1.652 | 60.77 | < .001 | 5.22 | — | — |

***Note.*** *OR = odds ratio; CI = confidence interval. Reference categories: gender = women; financial literacy = lacking financial literacy.*

The complete model for block 2 was statistically significant, χ²(4) = 274.91, *p* < .001, Nagelkerke *R*² = .20, AIC = 1776.

**Table B3**

*Logistic regression of self-reported expected difficulties paying bills during the next two months, Block 3 (N = 2020)*

| Variable | *B* | Wald | *p* | OR | 95% CI lower | 95% CI upper |
| --- | --- | --- | --- | --- | --- | --- |
| Financial literacy | -0.468 | 10.49 | .001 | 0.63 | 0.47 | 0.83 |
| Psychological distress | 1.033 | 210.07 | < .001 | 2.81 | 2.44 | 3.23 |
| Gender | 0.272 | 4.05 | .044 | 1.31 | 1.01 | 1.71 |
| Monthly gross income | -0.127 | 22.89 | < .001 | 0.88 | 0.84 | 0.93 |
| Age | -0.020 | 22.52 | < .001 | 0.98 | 0.97 | 0.99 |
| Intercept | -0.039 | 0.02 | 0.879 | 0.96 | — | — |

***Note.*** *OR = odds ratio; CI = confidence interval. Reference categories: gender = women; financial literacy = lacking financial literacy.*

The complete model for block 3 was statistically significant, χ²(5) = 521.08 *p* < .001, Nagelkerke *R*² = .36, AIC = 1532.

**Table B4**

*Logistic regression of self-reported expected difficulties paying bills during the next two months, Block 4 (N = 2020)*

| Variable | *B* | Wald | *p* | OR | 95% CI lower | 95% CI upper |
| --- | --- | --- | --- | --- | --- | --- |
| Financial literacy | -0.412 | 7.93 | .005 | 0.66 | 0.50 | 0.88 |
| Psychological distress | 0.889 | 116.32 | < .001 | 2.43 | 2.07 | 2.86 |
| Problem-focused coping | 0.012 | 0.40 | .527 | 1.01 | 0.98 | 1.05 |
| Emotion-focused coping | -0.018 | 1.33 | .249 | 0.98 | 0.95 | 1.01 |
| Avoidant coping | 0.071 | 16.10 | < .001 | 1.07 | 1.04 | 1.11 |
| Gender | 0.187 | 1.82 | .177 | 1.21 | 0.92 | 1.58 |
| Monthly gross income | -0.132 | 24.14 | < .001 | 0.88 | 0.83 | 0.92 |
| Age | -0.016 | 13.33 | < .001 | 0.98 | 0.98 | 0.99 |
| Intercept | -1.010 | 4.46 | 0.035 | 0.36 | — | — |

***Note.*** *OR = odds ratio; CI = confidence interval. Reference categories: gender = women; financial literacy = lacking financial literacy.*

The complete model for block 4 was statistically significant, χ²(8) = 537.70, *p* < .001, Nagelkerke *R*² = .37, AIC = 1521.

The final regression model was statistically significant, χ²(9) = 544,51, *p* < .001, Nagelkerke *R²* = .37, AIC = 1516.

**Table B5**

*Logistic regression of self-reported expected difficulties paying bills during the next two months with GAD-7 (N = 2020)*

| Variable | *B* | Wald | *p* | OR | Lower 95% CI | Upper 95% CI |
| --- | --- | --- | --- | --- | --- | --- |
| Anxiety symptoms (GAD-7 total) | 0.12 | 66.88 | < .001 | 1.13 | 1.09 | 1.16 |
| Financial literacy | -0.44 | 9.02 | .003 | 0.65 | 0.49 | 0.86 |
| Problem-focused coping | 0.17 | 1.32 | .250 | 1.19 | 0.89 | 1.60 |
| Emotion-focused coping | -0.16 | 0.68 | .408 | 0.99 | 0.96 | 1.02 |
| Avoidant coping | 0.53 | 13.77 | < .001 | 1.70 | 1.29 | 2.26 |
| Loneliness | 0.03 | 12.63 | < .001 | 1.03 | 1.01 | 1.05 |
| Gender | 0.15 | 1.14 | .285 | 1.16 | 0.89 | 1.52 |
| Monthly gross income | -0.13 | 22.32 | < .001 | 0.88 | 0.84 | 0.93 |
| Age | -0.02 | 13.27 | < .001 | 0.98 | 0.98 | 0.99 |
| Intercept | -4.081 | 39.62 | < .001 | 0.02 | — | — |

***Note.*** *OR = odds ratio; CI = confidence interval. Reference categories: gender = women; financial literacy = lacking financial literacy.*

The regression model of self-reported expected difficulties paying bills during the next two months, containing only GAD-7 as a measurement of psychological distress, was statistically significant, χ²(9) = 532,15, *p* <.001, Nagelkerke *R²* = .36, AIC = 1529.

**Table B6**

*Logistic regression of self-reported expected difficulties paying bills during the next two months with PHQ-9 (N = 2020)*

| Variable | *B* | Wald | *p* | OR | Lower 95% CI | Upper 95% CI |  |
| --- | --- | --- | --- | --- | --- | --- | --- |
| Depression (PHQ-9) | 0.10 | 65.85 | < .001 | 1.11 | 1.08 | 1.13 | |
| Financial literacy | -0.41 | 7.84 | .005 | 0.66 | 0.50 | 0.88 | |
| Problem-focused coping | 0.19 | 1.53 | .215 | 1.21 | 0.90 | 1.62 | |
| Emotion-focused coping | -0.11 | 0.36 | .551 | 0.99 | 0.96 | 1.02 | |
| Avoidant coping | 0.50 | 12.15 | < .001 | 1.66 | 1.25 | 2.20 | |
| Loneliness | 0.02 | 8.70 | .003 | 1.03 | 1.01 | 1.04 | |
| Gender | 0.20 | 2.03 | .155 | 1.22 | 0.93 | 1.60 | |
| Monthly gross income | -0.13 | 23.73 | < .001 | 0.88 | 0.83 | 0.93 | |
| Age | -0.02 | 16.95 | < .001 | 0.98 | 0.97 | 0.99 | |
| Intercept | -3.998 | 38.16 | < .001 | 0.02 | — | — | |

***Note.*** *OR = odds ratio; CI = confidence interval. Reference categories: gender = women; financial literacy = lacking financial literacy.*

The regression model of self-reported expected difficulties paying bills during the next two months, containing only PHQ-9 as a measurement of psychological distress, was statistically significant, χ²(9) = 532,19, *p* <.001, Nagelkerke *R²* = .36, AIC = 1529.

**Table B7**

*Logistic regression of self-reported expected difficulties paying bills during the next two months with financial literacy as a continuous variable (N = 2020)*

| Variable | *B* | Wald | *p* | OR | Lower 95% CI | Upper 95% CI |
| --- | --- | --- | --- | --- | --- | --- |
| Financial literacy continous | -0.15 | 4.48 | .034 | 0.86 | 0.75 | 0.99 |
| Psychological distress | 0.80 | 78.39 | < .001 | 2.22 | 1.86 | 2.64 |
| Problem-focused coping | 0.19 | 1.58 | .209 | 1.21 | 0.90 | 1.63 |
| Emotion-focused coping | -0.17 | 0.82 | .366 | 0.99 | 0.96 | 1.02 |
| Avoidant coping | 0.48 | 10.79 | .001 | 1.61 | 1.21 | 2.15 |
| Loneliness | 0.02 | 6.79 | .009 | 1.02 | 1.01 | 1.04 |
| Gender | 0.17 | 1.47 | .226 | 1.18 | 0.90 | 1.55 |
| Monthly gross income | -0.13 | 22.01 | < .001 | 0.88 | 0.84 | 0.93 |
| Age | -0.02 | 13.35 | < .001 | 0.98 | 0.98 | 0.99 |
| Intercept | -2.063 | 9.02 | .003 | 0.13 | — | — |

***Note.*** *OR = odds ratio; CI = confidence interval. Reference categories: gender = women; financial literacy = lacking financial literacy.*

The regression model of self-reported expected difficulties paying bills during the next two months, containing financial literacy as a continuous variable, was statistically significant, χ²(9) = 540,73, *p* <.001, Nagelkerke *R²* = .37, AIC = 1520.

**Table B8**

*Logistic regression of self-reported expected difficulties paying bills during the next two months with additional control variables (N = 2020)*

| Variable | *B* | Wald | *p* | OR | 95% CI Lower | 95% CI Upper |
| --- | --- | --- | --- | --- | --- | --- |
| Financial literacy | -0.374 | 6.06 | .014 | 0.69 | 0.51 | 0.93 |
| Psychological distress | 0.764 | 67.42 | < .001 | 2.15 | 1.79 | 2.58 |
| Problem-focused coping | 0.022 | 1.23 | .268 | 1.02 | 0.98 | 1.06 |
| Emotion-focused coping | -0.017 | 1.10 | .295 | 0.98 | 0.95 | 1.02 |
| Avoidant coping | 0.057 | 9.24 | .002 | 1.06 | 1.02 | 1.10 |
| Loneliness | 0.021 | 5.70 | .017 | 1.02 | 1.00 | 1.04 |
| Gender | 0.207 | 2.10 | .148 | 1.23 | 0.93 | 1.63 |
| Monthly gross income | -0.162 | 19.48 | < .001 | 0.85 | 0.79 | 0.91 |
| Age | -0.012 | 3.03 | .082 | 0.99 | 0.98 | 1.00 |
| Employed full-time/part-time | 0.501 | 2.23 | .135 | 1.65 | 0.86 | 3.18 |
| Student | 0.384 | 1.16 | .281 | 1.47 | 0.73 | 2.95 |
| Unemployed | 0.664 | 3.87 | .049 | 1.94 | 1.00 | 3.77 |
| Retired | 0.081 | 0.04 | .840 | 1.09 | 0.49 | 2.38 |
| Disability/activity benefits | 0.256 | 0.47 | .492 | 1.29 | 0.62 | 2.68 |
| Other employment status | 0.068 | 0.02 | .884 | 1.07 | 0.43 | 2.66 |
| Partner, no children | -0.410 | 3.87 | .049 | 0.66 | 0.44 | 1.00 |
| Single, no children | -0.180 | 0.99 | .321 | 0.84 | 0.59 | 1.19 |
| Single, with children | 0.693 | 7.85 | .005 | 2.00 | 1.23 | 3.25 |
| Living with parents | -1.034 | 8.30 | .004 | 0.35 | 0.18 | 0.72 |
| Upper secondary school | -0.161 | 0.35 | .553 | 0.85 | 0.50 | 1.45 |
| Higher vocational education | 0.337 | 1.33 | .249 | 1.40 | 0.79 | 2.49 |
| University/College | -0.266 | 0.91 | .339 | 0.77 | 0.44 | 1.32 |
| Intercept | -2.253 | 7.75 | .005 | 0.11 | — | — |

***Note****. OR = odds ratio; CI = confidence interval. Reference categories: gender = women; financial literacy = lacking financial literacy; education = primary education; household composition = living with partner and children; employment status = option not chosen.*

The regression model of self-reported expected difficulties paying bills during the next 2 months, using household composition, education and employment status as additional controls was statistically significant. χ²(22) = 591.77, *p* < .001, Nagelkerke *R²* .40 = . AIC = 1495.
